# Acute compartment syndrome as the initial manifestation of chronic-phase chronic myeloid leukemia: a case report and review of the literature

**DOI:** 10.1186/s13256-016-0985-5

**Published:** 2016-07-21

**Authors:** Yoshikazu Nagase, Shuji Ueda, Hitomi Matsunaga, Aya Yoshioka, Yoshiyuki Okada, Tomohisa Machida, Keiichi Nakata, Fuka Mima, Risato Takeda, Daisuke Hayashi, Sadaharu Iio, Kohei Okita, Hiroyuki Narahara, Yuichi Yasunaga, Yoshiaki Inui, Sumio Kawata

**Affiliations:** 1 Department of Internal Medicine, Hyogo Prefectural Nishinomiya Hospital, 13-9, Rokutanji-cho, Nishinomiya, Hyogo 662-0918 Japan; 2 Department of Clinical Laboratory, Hyogo Prefectural Nishinomiya Hospital, Nishinomiya, Hyogo Japan; 3 Department of Orthopaedic Surgery, Hyogo Prefectural Nishinomiya Hospital, Nishinomiya, Hyogo Japan

**Keywords:** Acute compartment syndrome, Chronic myeloid leukemia, Thrombocytosis, Bleeding complications

## Abstract

**Background:**

Acute compartment syndrome is an orthopedic emergency requiring urgent fasciotomy to prevent irreversible damage. In hematological malignancies, acute compartment syndrome caused by severe soft tissue bleeding is extremely rare. We present a patient with chronic-phase chronic myeloid leukemia who had acute compartment syndrome caused by severe soft tissue bleeding in her right forearm.

**Case presentation:**

A 72-year-old Japanese woman was referred to our hospital with swelling and pain of her right forearm without a previous history of trauma. She was diagnosed with chronic-phase chronic myeloid leukemia. Extreme thrombocytosis was present, although no evidence of acquired von Willebrand disorder was found. Compartment syndrome caused by soft tissue bleeding was confirmed. An emergency fasciotomy for decompression was conducted. However, sustained postoperative bleeding occurred and required massive red cell concentrate transfusion. As her platelet count decreased by cytoreductive therapy, complete hemostasis was achieved.

**Conclusions:**

Patients with an extremely high platelet count might be at high risk for severe bleeding complications even without acquired von Willebrand disease. For the control of severe bleeding complications in patients with myeloproliferative disorder, the importance of thrombocyte reduction should be recognized.

## Background

Acute compartment syndrome (ACS) most often develops after significant traumas, particularly those involving long bone fractures. This syndrome may also occur following non-traumatic causes, although such incidents are less frequent. ACS caused by leukemic infiltration of the muscles is uncommon but has sometimes been documented in acute leukemia or non-Hodgkin lymphoma [1–4]. It is rare for ACS to occur in the chronic phase of chronic myeloid leukemia (CML). Here we present a case of ACS that resulted from severe soft tissue bleeding in a patient with CML in the chronic phase.

## Case presentation

**Fig. 1:**
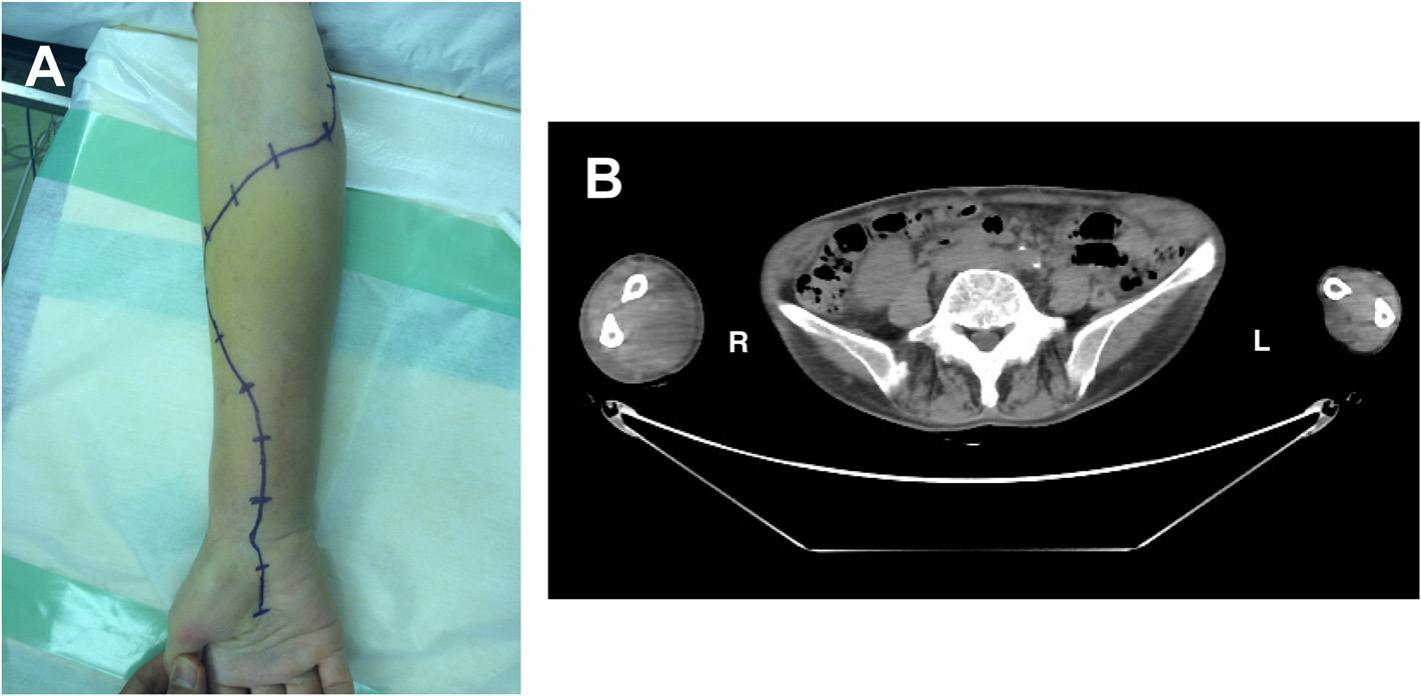
**a** Swollen right forearm of the patient. **b** Computed tomography revealing marked swelling of the right forearm compared with the left forearm

**Fig. 2:**
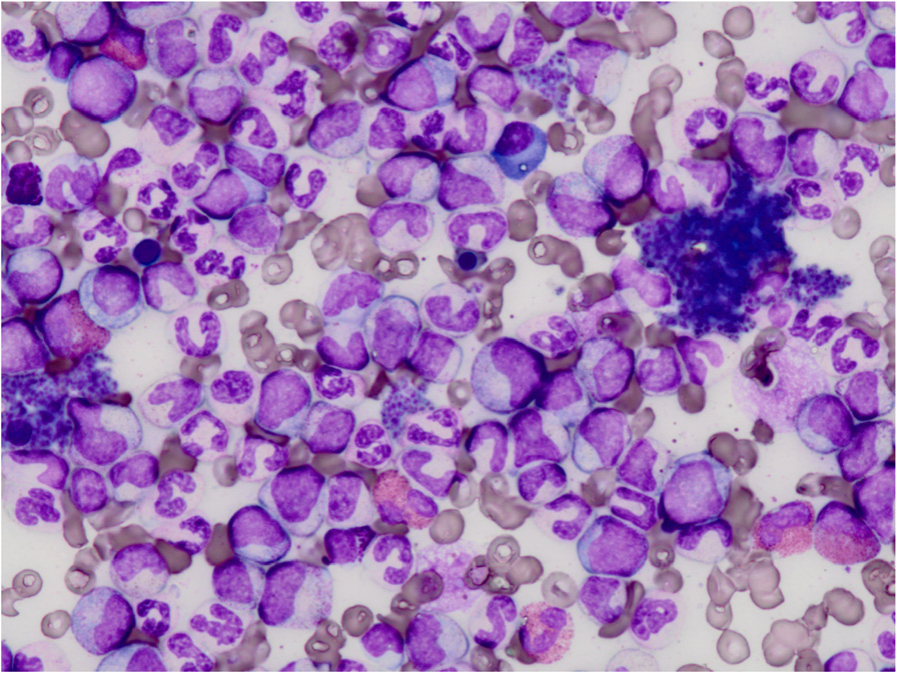
Bone marrow aspirate (May-Giemsa staining) showing marked myeloid proliferation without any differentiation block

A 72-year-old Japanese woman was referred to our hospital by her general practitioner with a 10-day history of swelling and pain in her right forearm without a previous history of trauma. A physical examination revealed that her right forearm was tense and swollen from the area directly proximal to her wrist to immediately below her elbow (Fig. 1a). She complained of increasing pain with passive extension and slight numbness of her fingers. Computed tomography revealed a low-density area in the muscles of the anterior aspect of her right forearm (Fig. 1b). Laboratory investigation showed a white cell count of 184.5 × 10^9^/L, including 1 % blast cells, 2 % eosinophils, 6.5 % basophils, 2.5 % promyelocytes, 14 % myelocytes, 18 % metamyelocytes, 44 % neutrophils, 7.5 % monocytes, and 4.5 % lymphocytes. In addition, her hemoglobin level was 66 g/L, and her platelet count was 3610 × 10^9^/L. Routine coagulation parameters revealed slightly prolonged prothrombin time (15.9 seconds, international normalized ratio 1.28). However, her activated partial thromboplastin time (32.6 seconds) was within the normal range of 24.0 to 35.0 seconds, as was her bleeding time (90 seconds). Plasma fibrinogen and D-dimer levels were slightly elevated to 390.8 mg/dL and 3.3 μg/mL, respectively (normal 200 to 380 mg/dL and 0 to 1.0 μg/mL, respectively), although her fibrin degradation product level was within normal range (8.2 μg/mL). The von Willebrand factor (vWF) antigen level was normal (111 %), and the vWF ristocetin cofactor activity, at 70 %, was undiminished. Multimeric analysis of vWF did not show a decrease of large vWF multimers. Furthermore, factor 8 and 13 activities, at 82 % and 72 % respectively, were not reduced. Laboratory values are summarized in Table 1. She did not have any apparent skin and mucosal bleeding tendency such as petechial hemorrhage. Bone marrow aspiration revealed hypercellularity with marked myeloid proliferation, but only 3 % of the cells were blasts (Fig. 2). These findings led to the presumption of a myeloproliferative disorder (MPD) such as CML, polycythemia vera, or essential thrombocytosis (ET). Because the level of her neutrophil alkaline phosphatase score was markedly reduced to 49 (normal 150 to 350), we deemed CML to be the most likely diagnosis.

**Table 1:** Laboratory values on admission

	Values	References
White cell count (10^9^/L)	184.5	35–91
NAP score	49	150–350
Hemoglobin (g/L)	66	113–152
Hematocrit (%)	23.2	33.4–44.9
Platelet count (10^9^/L)	3610	130–369
Bleeding time (seconds)	90	0–300
Prothrombin time (seconds)	15.9	10.5–13.5
INR	1.28	0.9–1.1
aPTT (seconds)	32.6	24–35
Fibrinogen (mg/dL)	390.8	200–380
FDP (μg/ml)	8.2	0–10
D-dimer (μg/ml)	3.3	0–1
Antithrombin III (%)	118.7	70–120
Factor VIII activity (%)	82	60–150
Factor XIII activity (%)	72	70–140
vWF: antigen (%)	111	50–150
vWF: Rco activity (%)	70	60–170
LDH (IU/L)	565	106–211
Albumin (g/dL)	4.0	4–5
Creatinine (mg/dL)	0.54	0.4–0.9
C-reactive protein (mg/dL)	0.23	0–0.3

*aPTT* activated partial thromboplastin time, *FDP* fibrin degradation product, *INR* international normalized ratio, *LDH* lactate dehydrogenase, *NAP* neutrophil alkaline phosphatase, *Rco* ristocetin cofactor, *vWF* von Willebrand factor

Cytoreductive therapy with hydroxyurea (1500 mg/day) was initiated as the most credible therapy to reduce her platelet count, because we could not rule out the possibility of MPD which does not respond to tyrosine kinase inhibitors. An urgent orthopedic opinion was obtained, and compartment syndrome was confirmed after the compartment pressure was found to be 39 mmHg. Emergency fasciotomy for decompression of the anterior compartment was conducted, which confirmed a tense compartment and pathologically proven hematoma in the right anterior compartment. There was no evidence of leukemic cell infiltration.

Persistent local bleeding developed during the latter part of the operation despite no surgical cause. After decompression, the wound was left open and managed with a wet dressing. Sustained postoperative oozing of blood resulted in hypovolemic shock and required massive red cell concentrate transfusion for hematologic and cardiovascular resuscitation. She continued to require 4 to 8 units of red blood cell concentrate on a daily basis (in Japan, 1 unit of red cell concentrate is derived from 200 mL of donated whole blood).

**Fig. 3:**
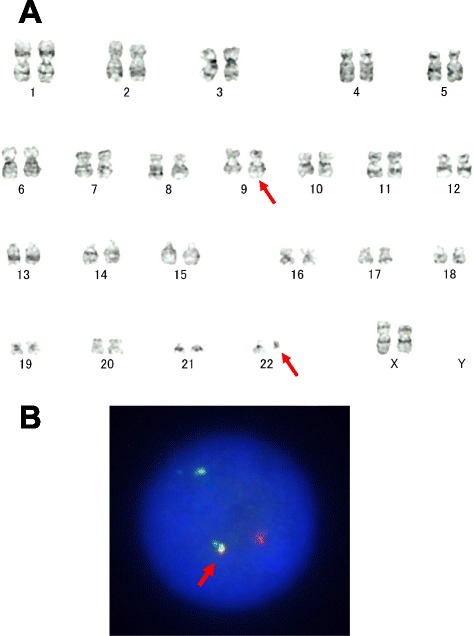
**a** G-banded karyotype showing 46,XX,t(9;22)(q34;q11.2). *Red arrows* indicate involved chromosomes 9 and 22. **b** Fluorescence *in situ* hybridization using the Vysis Extra Signal probe showing the *BCR–ABL* fusion signal. *Red arrow* indicates a red–green fusion (*yellow*) signal which confirms a *BCR* (*green*)/*ABL* (*red*) translocation

Chromosome analysis of her bone marrow cells demonstrated a karyotype of 46,XX,t(9;22)(q34;q11.2) (Fig. 3a). In addition, fluorescence *in situ* hybridization detected a *BCR–ABL* fusion signal in 93 % of the cells (Fig. 3b). Thus, she was diagnosed with chronic-phase CML [5]. Following this definitive diagnosis, dasatinib therapy (100 mg/day) was initiated. Although there were no signs of improvement in the oozing of blood while her platelet count remained high, her bleeding tendency began to improve after her platelet count decreased to less than approximately 1000 × 10^9^/L. During her clinical course her coagulation parameters remained stable. Although her D-dimer level, which reflects the dissolution of blood clots, remained slightly elevated (0 to 3 μg/mL) for a period of time, her prothrombin time and fibrinogen levels soon recovered to their normal range. Complete hemostasis was achieved 7 days after the fasciotomy, and the fasciotomy site was closed on postoperative day 20.

She continued dasatinib therapy, with good hematological and molecular responses. She obtained complete cytogenetic response and major molecular response at 12 and 18 months, respectively, after the initiation of dasatinib administration.

## Discussion

With patients with MPD, common complications involve gastrointestinal bleeding or skin and mucosal bleeding [6, 7]. Although most bleeding complications are generally not severe in patients with MPD [8], some do experience more severe symptoms [6].

In our patient, an extremely high platelet count (>1000 × 10^9^/L) and severe bleeding complications were observed. According to the *World Health Organization Classification of Tumours: Pathology and Genetics of Tumours of Haematopoietic and Lymphoid Tissues* (fourth edition), accelerated-phase CML is defined by the presence of one or more of six specific features including persistent thrombocytosis (>1000 × 10^9^ cells/L) that is unresponsive to therapy [5]. In our patient, her platelet count was reduced in response to a tyrosine kinase inhibitor, and this response was sustained. The diagnosis of chronic-phase CML was considered appropriate.

ACS is a limb-threatening and life-threatening emergency resulting from elevated intracompartmental pressure. ACS most often develops soon after significant traumas, but might arise as a complication of an underlying disease, such as soft tissue bleeding. In hematological malignancies, the causes of ACS are divided into two types: tumor cell infiltration or soft tissue bleeding. There are some case reports of ACS caused by tumor cell infiltration [1–4]. However, to the best of our knowledge, there have been only three case reports of ACS caused by soft tissue bleeding associated with hematological malignancies (Table 2). ACS caused by malignant cell infiltration has been reported in cases of acute leukemia or non-Hodgkin lymphoma [1–4]. In contrast, all three cases of ACS caused by soft tissue bleeding were patients with MPD. One case involved a patient with ET with extreme thrombocytosis [9]. In another case, thrombocytosis was not observed, but marked reduction of platelet aggregation function was present [10].

**Table 2:** Acute compartment syndrome caused by soft tissue bleeding related with hematological malignancies

Case	Age/Sex	Location	Disease	Platelet count (10^9^/L)	Operation (fasciotomy)	Other treatment	Reference
1	86/F	Left forearm	ET	3390	–	Plateletpheresis	[9]
2	32/M	Left thigh	CML (CP)	142	+	Activated recombinant factor VII	[10]
3	11/M	Left calf	CML (CP)	ND	+		[20]
Present case	72/F	Left forearm	CML (CP)	3610	+		

*CML* chronic myeloid leukemia, *CP* chronic phase, *ET* essential thrombocytosis, *F* female, *M* male, *ND* not described

Elevated platelet count is regarded as a risk factor for bleeding and thromboembolic events in MPD, but the significance of a high platelet count has not been confirmed in clinical studies [6]. Michiels *et al*. reported that hemorrhagic complications more often occur in patients with higher degrees of thrombocytosis than in those with lower degrees [11, 12]. Platelet dysfunction due to acquired von Willebrand syndrome (AvWS) is considered one of the reasons for bleeding tendency in patients with MPD with remarkable thrombocytosis [13, 14], owing to the increased number of platelets binding to highly prothrombotic ultra-large vWF and their removal from plasma [15]. However, neither decreased vWF levels (antigen and activity) nor an absence of higher molecular weight vWF multimers was confirmed in our patient. These results suggest that factors other than AvWS were responsible for the bleeding tendency of our patient.

Ristocetin-induced platelet aggregation abnormality is characteristic of AvWS platelet aggregation studies *in vitro*. In patients with MPD, various platelet function defects including platelet hyperactivity and hypoactivity, in addition to ristocetin-induced platelet aggregation, are frequently observed [16–18]. MPD-associated platelet dysfunction might cause bleeding complications. A patient with CML with platelet function abnormalities who developed severe soft tissue bleeding followed by compartment syndrome has been reported [10]; this patient did not experience AvWS-related complications. Following the administration of a tyrosine kinase inhibitor, platelet function improved together with the molecular response of CML. Moreover, in four patients with MPD with acute hemorrhagic or thrombotic complications of thrombocytosis, clinical symptoms improved with correction of platelet function after plateletpheresis [19]. These findings suggest that bleeding complications in patients with MPD could be improved by reducing the platelet count through correction of platelet dysfunction.

In the present case, prothrombin time was slightly prolonged, but activated partial thromboplastin time was within the normal range. Bleeding time was also normal, as measured using the Duke method. Our decision to perform the fasciotomy for decompression was based on these findings. However, sustained postoperative oozing occurred, and massive red cell transfusion was required. The cause of slightly prolonged prothrombin time on admission may be the decreased level of coagulation factor VII, although we did not investigate this. During our patient’s clinical course her prothrombin time quickly recovered to the normal range while oozing was still observed. Therefore, coagulation factor VII was not relevant to her bleeding tendency. *In vitro* platelet aggregation studies could not be conducted in our patient, but it is possible that her bleeding tendency might be caused by platelet dysfunction with MPD-associated thrombocytosis for the following three reasons: (i) although cessation of bleeding was not achieved while her platelet count remained high, hemostasis was achieved as her platelet count decreased; (ii) the abnormality of the coagulation parameters was imperceptible throughout her clinical course; and (iii) a patient with CML with platelet dysfunction reported by Ng *et al*. also showed a similar clinical course [10].

For the control of severe bleeding complications such as ACS in patients with MPD with thrombocytosis, it should be noted that bleeding tendency will probably last until the platelet count is sufficiently reduced. Because of hypovolemic shock due to sustained bleeding we hesitated to perform plateletpheresis. We should proactively consider treatment options such as plateletpheresis in addition to cytoreductive therapy before emergency fasciotomy.

## Conclusions

Patients with an extremely high platelet count might be at high risk for severe bleeding complications such as compartment syndrome even without acquired von Willebrand disease. For the control of severe bleeding complications in patients with MPD, the importance of thrombocyte reduction should be recognized.

## Abbreviations

ACS, acute compartment syndrome; AvWS, acquired von Willebrand syndrome; CML, chronic myeloid leukemia; ET, essential thrombocytosis; MPD, myeloproliferative disorder; vWF, von Willebrand factor
